# Partitioning of silver and chemical speciation of free Ag in soils amended with nanoparticles

**DOI:** 10.1186/1752-153X-7-75

**Published:** 2013-04-25

**Authors:** Rachel Benoit, Kevin J Wilkinson, Sébastien Sauvé

**Affiliations:** 1 Department of Chemistry, University of Montreal, Succ. Centre-Ville, P.O. Box 6128, Montreal, QC, H3C 3J7, Canada

**Keywords:** Chemical speciation, Complexation, Environmental fate, Ion selective electrode, Nanoparticles, Nanosilver, Contaminated soils

## Abstract

**Background:**

Knowledge about silver nanoparticles in soils is limited even if soils are a critical pathway for their environmental fate. In this paper, speciation results have been acquired using a silver ion selective electrode in three different soils.

**Results:**

Soil organic matter and pH were the most important soil properties controlling the occurrence of silver ions in soils. In acidic soils, more free silver ions are available while in the presence of organic matter, ions were tightly bound in complexes. The evolution of the chemical speciation of the silver nanoparticles in soils was followed over six months.

**Conclusion:**

During the first few hours, there appeared to be a strong sorption of the silver with soil ligands, whereas over time, silver ions were released, the final concentration being approximately 10 times higher than at the beginning. Ag release was associated with either the oxidation of the nanoparticles or a dissociation of adsorbed silver from the soil surfaces.

## Introduction

The field of nanotechnology has expanded rapidly in the last few years. As of March 2011, there were 1317 products or product lines containing nanomaterials [1]. Given its antimicrobial properties, nanosilver (nAg) is one of the nanomaterials found in the largest number of products [2] including consumer goods such as textiles, soaps and medical products [3,4]. In the environment, the main sources of nAg are expected to be from industrial wastes and consumer products. They are thought to enter the environment through sewage treatment plants, waste incineration plants and landfill [5-7]. Indeed, land application of sewage sludge as well as soil and water contamination from landfills are believed to be the most important contamination pathways for nAg. Nonetheless, most literature studies have examined the fate of nAg in water [8-10]. Much less attention has been given to soils, despite the importance of this pathway. Due to its small size, high reactivity and ill-defined dissolution properties, it is currently difficult to determine the environmental risks of nAg.

In natural waters, nAg has been shown to be toxic to several aquatic organisms [4,11,12]. In soils, toxicity tests have been performed with earthworms, however, the results appear to depend largely on the soil type. For example, for earthworms in a sandy loam soil [13-15], a small amount of nAg bioaccumulation in addition to reproductive toxicity and decreased growth were observed. For the soil nematode, *Caenorhabditis elegans*, reproduction was decreased by the addition of nAg [16]. Nonetheless, more controlled studies are required to evaluate the risks of nAg under conditions that are typical of natural soils. Specifically, given their ability to oxidize under environmentally-relevant conditions [14,17], it is not clear whether the nAg, Ag^+^ or Ag complexes are the most bioavailable species. Both Ag^+^ ions [18] and some of their complexes are known to affect bioavailability [19] or be toxic. Speciation studies are thus required in order to evaluate the risk of the nAg, and to distinguish it from that of the released free Ag and silver complexes. Some of the dissolved complexes of silver observed in soils include Ag_2_S, AgI, AgBr, AgCl, AgNO_3_, Ag_2_SO_4_ as well as dissolved organic matter [20].

In soils, free Ag^+^ is expected to preferentially bind to thiol groups and natural organic matter (NOM) [21]. Indeed, biologically-available silver is estimated to be less than 5% of the total dissolved silver because of the strong binding capacities of the humic and fulvic acids [22]. Furthermore, much of the Ag(I) in soils will be bound to colloidal particles in the size range between 10 and 200 nm or adsorbed to the soils [23]. For example, Cornelis et al. found a median partition coefficient (K_d_) of 1791 L kg^-1^ for dissolved Ag, implying that most of the Ag was highly retained by the soil [17].

Very few data are available on the partitioning of nAg in soils or on the determination of the speciation of Ag^+^ in soils following the amendment of nAg. It becomes more difficult to extrapolate and understand the fate and potential transformation of nAg added to soils. Specifically, no standard method exists that allows the unambiguous discrimination of silver ions from nAg in soils. The analytical difficulties arise largely from the significant oxidation of nAg in addition to the strong binding of free Ag^+^ by natural colloids in a similar size range [23]. Furthermore, the speciation of free Ag^+^ and the transformations of nAg can be influenced by pH, ionic strength and the presence of dissolved ions and natural organic matter [17]. Specialized techniques such as ultracentrifugation and X-ray absorption spectroscopy have been used to determine the speciation of silver in soil extracts or soils [14,17,23]. Simpler techniques such as the ion selective electrode (ISE) have been applied successfully to other metals in soil solutions [24,25]. The ISE has the ability to discriminate the free ion from its complexes, thus distinguishing it from other techniques that measure either dissolved silver or a proportion of the labile/dynamic metal complexes.

The aim of this study was to evaluate the chemical speciation free Ag^+^ and the sorption of silver nanoparticles in soils. The chemical speciation used focuses on the use of an Ag^+^ ion selective electrode and when combined with “total” solution measurements, can help us to differentiate what fraction of the nanoparticles would have dissolved and thus occur as free Ag^+^ ions and what portion would remain as nanoparticulate Ag or Ag associated with the colloids or soil solids. Both free (Ag ISE) and <0.45 μm fraction (by ICP-MS) were measured in three soils that were spiked with either nAg or silver nitrate. The evolution of the chemical speciation of free Ag^+^ was measured over time on one of the soils. The results provide insights into both the analytical determinations of Ag^+^ and the fate of nAg in soils.

## Materials and methods

### Soil samples

Two agricultural soils were collected from the Macdonald Campus Farm (Ste-Anne-de-Bellevue, QC, Canada) and one forest soil (Laval, QC, Canada). The surface layer (0–20 cm) was sampled and sieved to ≤2 mm. The physical and chemical characteristics of the soils are given in Table 1. Organic carbon was determined by dichromate redox titration method [26], cation exchange capacity (CEC) by BaCl_2_ method [27], pH using a soil paste H_2_O method [28] and particle size by a hydrometer method [29].

**Table 1:** Physical and chemical characteristics of the soils (averages are given with standard deviations of duplicate measurements)

**Soils**	**pH**	**Sand (%)**	**Silt (%)**	**Clay (%)**	**Organic carbon (%)**	**Conductivity (μS/cm)**	**Ionic Strength (mM)**	**CEC (cmol/kg)**
**Agricultural 1**	7.48	71.5	19.5	9	2.9±0.1	96±2	1.25	13.1±0.5
**Agricultural 2**	6.65	88	10	2	1.5±0.1	61±6	0.79	8.9±0.9
**Forest**	4.50	80	17	3	3.1±0.1	88.3±0.6	1.15	3.5±0.1

### Short-term exposures

The three soils were dried at 70°C overnight. In a 50-mL centrifuge tube, 20 mL of either a solution of silver nanoparticles (Vive Nano, now Vive Crop Protection) or silver nitrate were added to 10 g of the soils to obtain final nominal concentrations of 1, 5, 10, 25, 50, 100 mg Ag kg^-1^ dry soil. Sodium nitrate was added to the solutions (final concentration of 0.1 M) to facilitate measurements with the ISE. Solutions were shaken for 24 h on a reciprocal shaker in order to allow the system to approach equilibrium. The supernatant was isolated by centrifuging the samples for 10 min at 4500 g.

### Long-term exposures

Agricultural soil 2 was also exposed to silver nanoparticles for up to 200 days. In that case, the soil was dried at 70°C overnight prior to the addition of nAg or silver nitrate in order to obtain final nominal concentrations of 1, 5, 10, 25, 50, 100 mg Ag kg^-1^ (55 mL of silver solutions at 3,6; 18,2; 36,4; 90,9; 182; 364 mg L^-1^). Samples were shaken manually for 5 minutes. Two hundred grams of soil of each treatment were put in a pot covered with a geotextile membrane in order to prevent dust deposition while allowing air and water exchange. The soil was maintained in the laboratory and sampled at different times. The only water added thorough the exposure was the water of the silver solution. At intervals, 10 g aliquots were extracted with 20 mL of 0.1 M NaNO_3_. The tubes were shaken vigorously for 20 min and then centrifuged at 4500 *g* for 10 min. The analytical data for the smaller spiked concentrations (1 to 10 mg Ag kg^-1^) were not consistent and only the higher concentrations are reported (25 to 100 mg Ag kg^-1^) see Additional file 1: Figure S1.

### ISE measurements

The pH and the free Ag^+^ were measured in the supernatants without prior filtration. Soil solution pH was measured with a calomel electrode (Accumet) and the free Ag^+^ was measured with a combined silver ISE (Orion 9616BNWP). The Ag ISE was calibrated daily with concentration of silver nitrate that varied between 1×10^-7^ M and 1×10^-3^ M. Measurements were made in order of increasing concentrations in order to avoid memory effects. Between samples, the electrode was rinsed with 10% v/v HNO_3_ and Milli-Q water (R>18 MΩ cm. total organic carbon<2 μg C L^-1^) and gently blotted with laboratory tissues.

Chemical speciation calculations for the calibration using buffers were made using the “Visual MINTEQ” software (Gustafsson, 2010) and the default constants for complexation with halogens (Cl, Br, I). For the calculation, 9,18×10^-6^ M of Ag^+^ was mixed with 0,1M NaCl or KBr while 9,26×10^-7^ M of Ag^+^ was mixed with 0,01 NaI. The stability and reproductibility of the free Ag^+^ ion selective electrode was evaluated.

### Characterization of the nAg

The nAg are stabilized by sodium polyacrylate (Ag content is 31% w/w) and were purchased from Vive Crop Protection (formerly Vive Nano, Toronto, Canada). Particle size distribution, as determined by transmission electron microscopy and fluorescence correlation spectroscopy, were between 1–10 nm [30].

### Chemical analysis

Following centrifugation of the soils, the supernatants were filtered with a 0.45 μm cellulose acetate filter membrane (Whatman). The concentration of Ag in the <0.45 μm fraction was determined by adding 2 mL of concentrated HNO_3_ to 2 mL of the supernatant and then digesting the sample for 48 hours at 98°C. This fraction is presumed to represent the sum of colloidal and dissolved Ag but could include also free Ag^+^ which is bound to colloids. The total silver content of the soil was determined by digesting 1 g of dry soil overnight (90°C) in 10 mL of concentrated nitric acid (Trace metal grade). The acid digests were filtered with a Q5 filter (Fisherbrand) and diluted to 50 mL. Acidified solutions were diluted appropriately for the analysis of Ag by either graphite furnace atomic absorption spectrometry (GFAAS; Varian) or by inductively coupled plasma mass spectrometry (ICP-MS; NexION 300×, Perkin Elmer).

### Quality control

Three replicates were measured for the short-term exposures and duplicate samples were analysed for the long-term exposures. Quality controls were performed in order to ensure the accuracy of the results. A sandy loam certified reference soil (CRM027-050), a non-spiked soil and a nitric acid control were analysed with each batch of samples. A certified reference material (NIST1640a) was analysed to verify the validity of the GF-AAS and ICP-MS analyses, measurements were within 15% of certified value (8.08 ± 0.05 μg L^-1^ for a sample reported to contain 8.4±0.5 μg/L).

## Results and discussion

**Figure 1:**
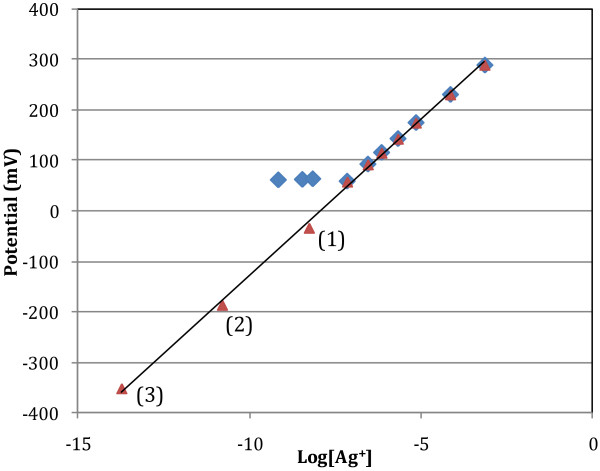
**ISE calibration curve for the measured potential of the Ag ISE as a function of the log[Ag**^**+**^**] activity.** (Blue diamonds): solutions of AgNO_3_ in 0.1 M NaNO_3_. (Red triangles): solutions of AgNO_3_ in 0.1 M NaNO_3_, except (**1**) 0.1M NaCl, (**2**) 0.1 M KBr, (**3**) 0.01 M NaI. The electrode response yielded a Nernstians response: Potential =61.6[Ag^+^]+490, R^2^=0.998.

The calibration of the ISE is shown in Figure 1. The limit of detection was 1X10^-7^ M when measurements were performed in Milli-Q water with added sodium nitrate (blue diamonds). However, following the addition of ligands for the Ag (Cl, Br, I), it was possible to calibrate the electrode down to 1×10^-14^ M Ag^+^ with an excellent linearity (R^2^=0.998) and a nearly theoretical Nernstian plot (slope=62 mV M^-1^ vs. 59 mV M^-1^).

The effect of changing ionic strength from 0.01 M, 0.05 M and 0.1 M NaNO_3_ had a minimal impact on the electrode response (calibration varying form 55×+525.0 to 56.2 × + 526.3 and 56.6 × + 526.1 – see Additional file 2: Figure S2).

The response of the ion selective electrode to free Ag^+^ has been surprisingly stable over 22 calibrations curves spawning 4 months of use (see Additional file 3: Figure S3).

**Figure 2:**
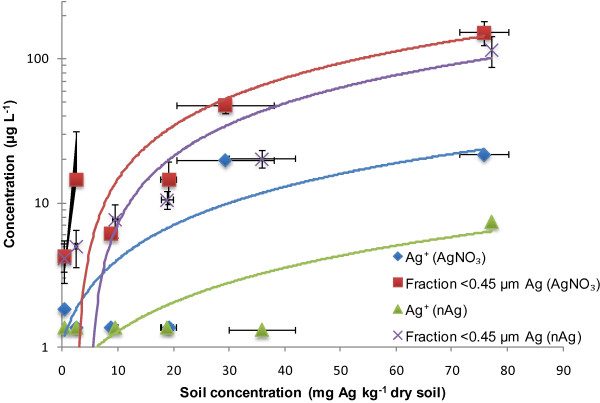
**Measurements of Ag**^**+ **^**and total solution Ag (presumed dissolved) following the addition of either nAg and AgNO**_**3 **_**in Agricultural soil 1.** The blue diamonds represent free Ag^+^ for the AgNO_3_ treatment, the red squares are for the <0.45 μm fraction in the AgNO_3_ treatment, the green triangles are for free Ag^+^ for the nAg treatment and the purple X are for the <0.45 μm fraction in the nAg treatment.

**Figure 3:**
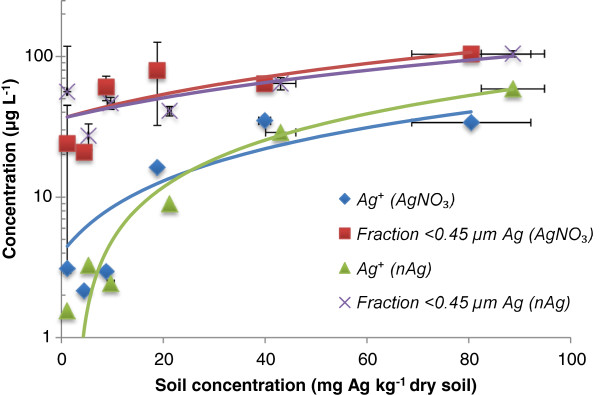
**Measurements of Ag**^**+ **^**and total solution Ag (presumed dissolved) following the addition of either nAg and AgNO**_**3 **_**in Agricultural soil 2.** The blue diamonds represent free Ag^+^ for the AgNO_3_ treatment, the red squares are for the <0.45 μm fraction in the AgNO_3_ treatment, the green triangles are for free Ag^+^ for the nAg treatment and the purple X are for the <0.45 μm fraction in the nAg treatment.

**Figure 4:**
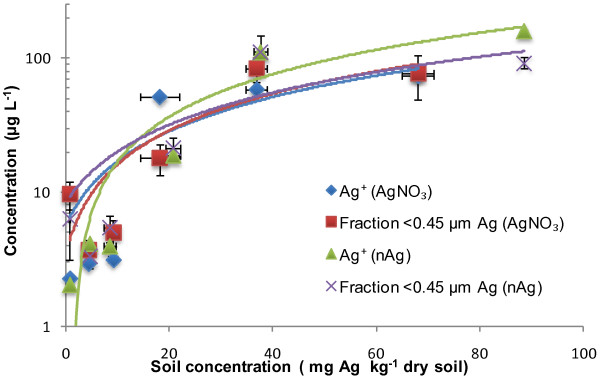
**Measurements of Ag**^**+ **^**and total solution Ag (presumed dissolved) following the addition of either nAg and AgNO**_**3 **_**in the Forest Soil.** The blue diamonds represent free Ag^+^ for the AgNO_3_ treatment, the red squares are for the <0.45 μm fraction in the AgNO_3_ treatment, the green triangles are for free Ag^+^ for the nAg treatment and the purple X are for the <0.45 μm fraction in the nAg treatment.

The retention and chemical speciation of silver in the 3 soils are shown in Figures 2, 3 and 4. For treatments of either Ag nitrate or nAg, 3 measurements were performed: (i) total Ag measured in the soil (x axis), (ii) <0.45 μm fraction, which presumably refelects the sum of dissolved and colloidal Ag and (iii) free Ag^+^ in solution. Generally speaking, the data do not necessarily behave as expected from standard Freundlich-type sorption. The AgNO_3_ treatments generated higher Ag concentrations in the <0.45 μm solution fraction than did an equivalent treatment of nAg (i.e. spiking to similar total soil Ag levels). Furthermore, less free Ag^+^ in solution was observed in Agricultural soil 1 as compared to the other two soils. We attribute this result to the relatively high pH of this soil (pH 7.48) and the fact that it contains twice the concentration of organic matter with respect to Agricultural soil 2 and a higher clay content. At higher pH values, free Ag^+^ is expected to be increasingly sorbed onto the mineral surfaces of the soil, since at higher pH, there is a higher CEC and thus more negatively charged surface sites [22]. Also, free Ag^+^ binds tightly to organic matter, which could explain why we see less free Ag^+^ in this soil with respect to the other soils. Silver has a strong affinity for the nitrogen and the sulphur groups that can be found in natural organic matter [31,32].

In contrast, in Agricultural soil 2 (Figure 4), there are only slight, potentially insignificant, differences between the concentrations of Ag in the <0.45 μm fraction observed following equivalent treatments of AgNO_3_ and nAg. These data suggest that the nAg was nearly completely dissolved. An alternative hypothesis presuming that the Ag^+^ and nAg were adsorbed in a similar manner to the soil is unlikely since it assumes that the nAg would not significantly oxidize. The major difference between the two soils was their pH (7.48 in soil 1 vs. 6.65 in soil 2) and their organic matter content (2.9%C in soil 1 vs. 1.5%C in soil 2). The higher pH and higher organic matter content in soil 1 could help stabilize the nAg [15], since lower pH favors the oxidation of the nAg [9]. In both soils, their circumneutral pH favours both the adsorption of the free Ag^+^ and its complexation to dissolved organic matter in solution, thus explaining why there was a significant difference between the <0.45 μm fraction and free Ag^+^ in solution.

For the addition of Ag to the acidic (pH 4.5) forest soil, the four curves were nearly superimposed, suggesting that most of the nAg was dissolved and non-complexed. In this case, the acidic soil solution likely both promoted the oxidation of the nAg and minimized free Ag^+^ sorption onto the soil solids (Figure 4). The lower pH is also partly responsible for the low CEC observed in Table 1. In the Forest soil, most of the silver in solution appeared to occur as free Ag^+^, consistent with a lower affinity of cations for the soil surfaces under acidic conditions [21].

The properties of the soils played an important role in the speciation of the nAg. For example, Shoults-Wilson et al. found that that the oxidation rate of nAg depended on the soil type [14]. In their case, sandier and acidic soils like the Forest soil showed a greater toxicity, with ions being more available for biological uptake. In contrast, for Agricultural soil 1, significant bioavailability of Ag would not be expected due to the strong binding of Ag by organic matter.

**Figure 5:**
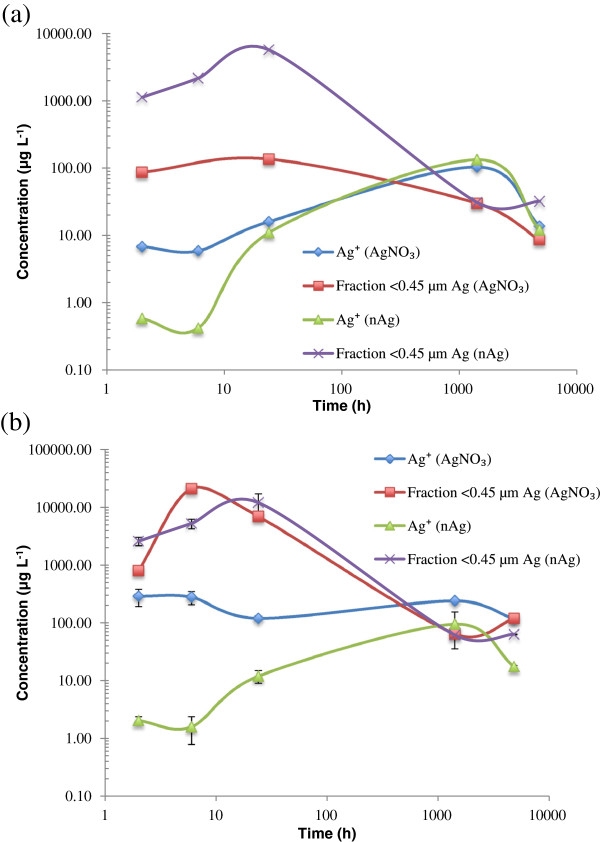
**Time evolution of silver fractionation.** Speciation measurements of Ag^+^ and total solution Ag (presumed dissolved) following the addition of either nAg and AgNO_3_ for a long-term exposure of Ag in agricultural soil 2 (**a**) 25 mg Ag/kg dry soil (**b**) 50 mg Ag /kg dry soil. The blue diamonds represent free Ag^+^ for the AgNO_3_ treatment, the red squares are for the <0.45 μm fraction in the AgNO_3_ treatment, the green triangles are for free Ag^+^ for the nAg treatment and the purple X are for the <0.45 μm fraction in the nAg treatment.

For the long-term exposures, the addition of 25–100 mg Ag kg^-1^ dry soil, as either AgNO_3_ or nAg, resulted in similar concentrations of free Ag^+^ in solution after circa a month of equilibration (Figure 5 and Additional file 1: Figure S1). We can speculate that the Ag was rapidly bound to the available ligands in the soil solution or onto soil surfaces during the first hours of exposure. Subsequently, as the system equilibrates, Ag dissociation from the soils may have resulted in higher free Ag^+^. For example, Courtris et al. demonstrated that uncoated NPs were a constant source of bioavailable Ag [23]. For the same time series, a reverse trend was observed for the Ag concentrations in the <0.45 μm fraction: adding 25 mg Ag kg^-1^ as nAg initially generated a much higher concentration of dissolved Ag than adding an equivalent amount of AgNO_3_ (see Figure 5a). In this case, the nAg are coated with sodium polyacrylate and it is possible that over time, the coatings are destabilized and the Ag oxidized, thus releasing free Ag^+^ which was then able to sorb onto the soils.

For the addition of a higher concentration of total soil Ag (50 mg kg^-1^ dry soil), a different behaviour was observed (Figure 5b). In this case, the addition of such large quantities of Ag probably exceeded the sorption capacity of the soil and may have generated higher free Ag^+^ in solution when compared to the addition of an equivalent concentration of nAg. Figure 5b also illustrated that with respect to the concentration of Ag in the <0.45 μm fraction, when the sorption capacity of the system was saturated, it made no difference as to whether AgNO_3_ or nAg was added.

The patterns observed in Figure 5 and Additional file 1: Figure S1 for AgNO_3_ and nAg were similar. The concentration of Ag in the <0.45 μm fraction was higher than free Ag^+^ during the first hours of exposure but the concentrations were similar after 6 months. After 6 months, the concentration of Ag remaining in the <0.45 μm fraction seemed to correspond mostly to free Ag^+^. Also, the concentration of Ag in the <0.45 μm fraction for the nAg treatment was higher than that observed for the AgNO_3_ treatment, especially for the soil having received 25 mg kg^-1^ (Figure 5a). This could probably be explained by a slower dynamics of the nanoparticles in the soil as compared to AgNO_3_[23].

## Conclusion

This study provides a method to differentiate free silver ions from silver in the <0.45 μm fraction in soil solution and helps us understand how nAg reacts in soils. It indicated that nAg is likely to dissolve under normal soil conditions and that the coating of nanoparticulate Ag and the physicochemical properties of the soils are likely very important when determining the integrity, retention and mobility of silver nanoparticles. The roles of organic matter and pH are likely key factors controlling the transformation of nAg amendments to soils. More studies need to be performed on more types of soils in order to fully understand the chemical speciation and fate of nAg.

## Competing interests

The authors declare they have no competing interests.

## Authors’ contributions

RB has realized most of the laboratory manipulations and data treatment. The experimental setup was designed by all co-authors (RB, KW & SS) and RB has written the initial version of the manuscript which KW and SS have further commented and reviewed. All authors have read and approved the final manuscript.

## Supplementary Material

Additional file 1: Figure S1 Long-term exposure of Ag in agricultural soil 2 spiked at 100 Ag mg kg^-1^ dry soil. Click here for file

Additional file 2: Figure S2 Calibration of the silver specific electrode depending on the ionic strengh (**a**) 0,01 M NaNO_3_ (**b**) 0,05 M NaNO_3_ (**c**) 0,1 M NaNO_3._ Click here for file

Additional file 3: Figure S3 Response of the silver ionic electrode over 4 months of use. Click here for file
