# Supplementary material for: Partitioning of silver and chemical speciation of free Ag in soils amended with nanoparticles
Source: Chem Cent J. 2013 Apr 25;7:75. doi: 10.1186/1752-153X-7-75 (PMC3648414; doi:10.1186/1752-153X-7-75)
Supplement: Additional file 1: Figure S1 — Long-term exposure of Ag in agricultural soil 2 spiked at 100 Ag mg kg-1 dry soil. [file 1752-153X-7-75-S1.docx]

**Figure S1** Speciation measurements of free Ag^+^ and solution Ag in the <45 µm fraction (presumably the sum of dissolved and colloidal Ag) following the addition of either nAg and AgNO_3_ for a long-term exposure of Ag in agricultural soil 2 spiked at 100 Ag mg kg^-1^ dry soil.
