# Supplementary material for: Partitioning of silver and chemical speciation of free Ag in soils amended with nanoparticles
Source: Chem Cent J. 2013 Apr 25;7:75. doi: 10.1186/1752-153X-7-75 (PMC3648414; doi:10.1186/1752-153X-7-75)
Supplement: Additional file 2: Figure S2 — Calibration of the silver specific electrode depending on the ionic strengh (a) 0,01 M NaNO3 (b) 0,05 M NaNO3 (c) 0,1 M NaNO3. [file 1752-153X-7-75-S2.docx]

(a)

(b)

(c)

**Figure S2** Calibration of the silver specific electrode depending on the ionic strengh (a) 0,01 M NaNO_3_ (b) 0,05 M NaNO_3_ (c) 0,1 M NaNO_3_.
