# Supplementary material for: Partitioning of silver and chemical speciation of free Ag in soils amended with nanoparticles
Source: Chem Cent J. 2013 Apr 25;7:75. doi: 10.1186/1752-153X-7-75 (PMC3648414; doi:10.1186/1752-153X-7-75)
Supplement: Additional file 3: Figure S3 — Response of the silver ionic electrode over 4 months of use. [file 1752-153X-7-75-S3.docx]

**Figure S3** Response of the silver ionic electrode over 4 months of use.
